# Heart rate synchrony as an objective biomarker in psychotherapy: a wearable-based pilot study

**DOI:** 10.1007/s00406-026-02248-4

**Published:** 2026-05-19

**Authors:** Clara C. Gernert, Christine M. Falter-Wagner

**Affiliations:** 1 Department of Psychiatry and Psychotherapy, LMU University Hospital, Nussbaumstr. 7, 80336 Munich, Germany; 2 German Centre for Mental Health (Deutsches Zentrum für Psychische Gesundheit, DZPG), Site Munich-Augsburg, Munich, Germany

**Keywords:** Psychotherapy research, Heart rate, Interpersonal synchrony, Wearable technology, Physiological coupling, Therapeutic alliance

## Abstract

Therapeutic alliance is a well-known predictor of psychotherapy outcome and is often measured by subjective self-reports prone to biases. This pilot study examined whether interpersonal synchrony (IPS) in movement and heart rate (HR), derived from wearable wristbands, can serve as an objective measure of therapeutic engagement in naturalistic cognitive behavioral therapy sessions. Twelve transdiagnostic patient-therapist dyads were analyzed in a short-term follow-up design. Physiological signals were continuously recorded with multisensor wrist-worn devices, while head and body movements were extracted from video recordings. After each session, both dyad members completed alliance questionnaires, and patients provided standardized symptom ratings. We quantified IPS for both modalities by lagged-windowed cross-correlations. In-phase HR synchrony during the initial session significantly predicted changes in patients’ global severity index and depressive symptomatology over time. Notably, only therapists’ alliance ratings were associated with short-term outcomes. While HR data obtained from wristband sensors show potential for assessing interpersonal physiological coupling, results remain preliminary given the small, artifact-filtered subsample analyzed. The convenience and unobtrusive nature of wearable technology allows low-threshold collection of objective data even in the sensitive setting of psychotherapy. However, this pilot study indicates that convenience has to be weighed against data quality. Achieving higher precision with high-resolution devices may require greater intrusiveness. Establishing more consistent methodological standards are essential to advance IPS-based approaches towards characterizing and individualizing evidence-based psychotherapy.

**Supplementary Information:**

The online version contains supplementary material available at 10.1007/s00406-026-02248-4.

## Introduction

In light of the high prevalence rates of mental disorders [1–3], as well as their risk of chronic development and symptom aggravation if left untreated, there is a growing need to identify early, valid predictors of psychotherapeutic outcomes. This need is further exacerbated by the limited availability of psychotherapy as a treatment option. According to Grawe’s neuropsychological theory, mental disorders may be conceptualized as the outcome of repeated failures in the regulation of internal inconsistency or incongruence of motivation [4]. The primary therapeutic aim in this line of thinking is to restore functional consistency through emotionally and cognitively corrective experiences within the therapeutic process [4]. Psychotherapy itself constitutes a complex bio-psycho-social system in which patterns of language, emotion, and cognition are continuously shaped and reshaped through reciprocal patient-therapist interactions [5]. The interaction itself is inherently dynamic, involving two interdependent systems, patient and therapist, which mutually influence and regulate each other in real-time, within the given asymmetric setting of psychotherapy [6]. Within this relationship, the co-occurrence of interpersonal and emotional features plays a pivotal role in forming the therapeutic alliance (TA), a construct widely acknowledged as critical for therapeutic effectiveness [e.g., 7–16]. Originally conceptualized by Bordin, the TA is characterized by three interrelated components: agreement on goals, consensus on tasks, and the development of an emotional bond [17]. Alliance scores obtained during the early and terminal stages of therapy have been found to predict outcomes more accurately than those measured during intermediate phases [18–20]. Furthermore, alliance ratings collected after the first session have been shown to predict premature termination [20–21], highlighting the prognostic value of early relational markers. However, concepts exist suggesting that therapeutic success is not only a result of working alliance quality per se but also depends on the individual characteristics of both therapist and patient [22–27].

While the TA was traditionally framed as a product of cognitive-emotional engagement and verbal exchange, recent theoretical and empirical research has emphasized the embodied dimension of TA. Interpersonal synchrony (IPS), defined as “the degree to which the behaviors in an interaction are non-random, patterned or synchronized in both timing [and] form.” [28, p. 403] reflects one such embodied mechanism. IPS is not limited to non-verbal behavior, such as movement [e.g. 29–31], but also manifests in speech [e.g. 32], physiology [33–36], and even brain activity [e.g. 37, 38]. Research has found IPS to be fundamental in fostering social relationships and cohesion by showing associations with rapport [e.g. 39, 40], affiliation [41], successful cooperation [e.g. 34, 42], prosocial commitment [43], and empathy [44]. Moreover, biobehavioral synchrony has been proposed as a mechanism for acquiring resilience [45].

As a measure of social connection, IPS may play a crucial role in psychotherapeutic treatment relationships [46]. While movement synchrony has been associated with therapy outcomes, physiological synchrony has been mostly linked to the concept of TA [46] and empathy [44]. Nevertheless, only a limited number of studies have examined physiological IPS in psychotherapy, and only a few of them used wearable devices. Physiological synchrony variables like HR dynamics [47, 35], respiration [35], and skin conductance [33, 44] were assessed mostly in small samples and using conventional, lab-based monitoring systems. Although HR is a well-established biomarker for affective and cognitive states, and a key index of sympathetic and parasympathetic arousal [48], only a few studies have explored HR synchrony in therapeutic settings. Studies have primarily focused on its correlation with subjective ratings such as alliance [35] or empathy [47], but less so on therapy outcome. While the TA is often assessed by subjective reports, thereby introducing potential inconsistencies and biases, wearable-based biosensing technology offers a promising tool for capturing objective, continuous, and ecologically valid physiological data.

Building on the *In-Sync* model [49], which proposes that IPS promotes affective co-regulation, and thereby TA, the current pilot study aimed to evaluate the presence and clinical relevance of HR synchrony in cognitive behavioral therapy (CBT). HR data from patients and respective therapists were continuously recorded using wristband sensors during two naturalistic CBT sessions each. Concurrently, body and head movements were captured via a stationary video camera. Following each CBT session, patients and therapists completed standardized post-session reports. Moreover, patients documented psychiatric symptoms and psychological distress via standardized questionnaires. Interpersonal HR and movement synchrony were analyzed both as indices of TA and as potential predictors of therapy outcome, operationalized as a patient-reported change in symptom severity over time. Specifically, the study addressed the following two research questions: (1) are IPS indices associated with alliance ratings, and (2) do IPS indices or alliance ratings predict therapy outcome.

## Materials and methods

### Participants

As a transdiagnostic pilot sample, 25 patient-therapist dyads were recruited from the Clinic for Psychiatry and Psychotherapy (LMU University Hospital, Munich). Exclusion criteria included cardiac disease or implants, acute substance abuse, high-dose beta-blockers, acute neurological disorders, or severe skin defects. All participants had normal or corrected-to-normal vision. The therapists were not involved with the research team, study design, or data analysis. Of the 25 recruited dyads, 19 dyads participated in a short-term follow-up design, comprising two recorded CBT sessions per dyad. The other six dyads completed only one session and were therefore excluded from analysis. Of the 19 dyads in the follow-up group, four dyads were removed from analysis due to technical failures during data acquisition in at least one of their two recorded sessions. Of the remaining 15 dyads, three dyads were excluded due to poor signal quality in the HR time series of at least one dyad member. The signal quality check was performed after converting and preprocessing the raw photoplethysmography (PPG) signals into HR time series (for details, see Sect.  3.2.1). As a result, 12 complete datasets were retained for statistical analysis. For each of these 12 dyads, at least two weeks passed between the initial and follow-up CBT sessions, with the second session typically taking place during the third week after the first. The follow-up group comprised a mixed clinical patient sample (*N* = 12, 66.7% female, 33.3% male, M_age_ = 35.1 ± 13.8) with their belonging therapist (N_Therapist_ = 8, 87.5% female, 12.5% male, M_age_ = 32.4 ± 8.6). The dyads’ gender composition was balanced, with six same-sex and six mixed-sex dyads. Each patient had at least one (*n* = 8) or several (*n* = 4) diagnoses of a mental disorder according to ICD-10, Chapter V(F) [50]. The descriptives are summarized in Tables 1, 2 and 3. Comparing inclusion and exclusion group, there was no significant difference in their descriptives (see Supplementary Material, Table 1). All participants provided written informed consent, and the study received IRB approval from the LMU Medical Faculty Ethics Board (19–170).

**Table 1:** Demographics and clinical characteristics

Variable	Category	Patients (*N* = 12)	Therapists (*N* = 8)
Age, mean ± SD (years)		35.1 ± 13.8	32.4 ± 8.6
Gender, n (%)	Female	8 (66.7)	7 (87.5)
	Male	4 (33.3)	1 (12.5)
	Diverse	0 (0.0)	0 (0.0)
Region of origin, n (%)	Europe	11 (91.7)	8 (100.0)
	Latin America	1 (8.3)	0 (0.0)
Smoking status, n (%)	Non smoker	9 (75.0)	
	Current smoker	3 (25.0)	
Alcohol use, n (%)	None	8 (66.7)	
	Social	2 (16.7)	
	Regular	2 (16.7)	
Illicit substance use, n (%)	None	10 (83.3)	
	Cannabinoids	2 (16.7)	
	Amphetamine	1 (8.3)	
Somatic comorbidities, n (%)	None	10 (83.3)	7 (87.5)
	Positive	2 (16.7)	1 (12.5)

Frequencies (*n*) and percentages are reported for patients’ and therapists’ descriptives. Age is reported as mean values in years with corresponding standard deviations (SD). In this sample, sex assigned at birth was consistent with gender identity and is therefore reported under the term *Gender*. Illicit substance use refers to past use of illegal substances. All participants included had been abstinent for several years. Acute substance use was an exclusion criterion. Smoking status and alcohol consumption were assessed for patients only. Participants with somatic comorbidities were not present with relevant cardiovascular or neurological disorders

**Table 2:** Patients’ psychiatric diagnoses and medication

Variable	Category	Frequency	*%* (*N* = 12)
ICD-10 F-codes	F1	1	8.3
	F2	0	0.0
	F3	9	75.0
	F4	4	33.3
	F5	0	0.0
	F6	3	25.0
Number of diagnoses	Single	8	66.7
	Multiple	4	33.3
Medication	None	1	8.3
	Antidepressants	10	83.3
	Antipsychotics	4	33.3
	Antiepileptics	1	8.3
	Anxiolytika	2	16.7
	Sedatives	2	16.7
	Sympathomimetics	2	16.7
	Low-dose beta-blocker	1	8.3
	Thyroid hormone	1	8.3

Absolute and relative frequencies of ICD-10 F-codes (World Health Organization, 1993) are reported. Percentages exceed 100% because some patients met criteria for more than one diagnostic category. The number of diagnoses refers to patients with single versus multiple current psychiatric diagnoses. Medication use is presented as absolute and relative frequency. Medications reflect regular treatment at the time of assessment. Only one patient was not taking any medication at all. Multiple medications per subject are possible

**Table 3:** Subclasses of antidepressants and augmentation strategies

Variable	Category	Frequency	%
AD class	SSRIs	4	
	SNRIs	2	
	Tricyclic ADs	1	
	Atypical ADs	5	
AD augmentation	Monotherapy	8	80.0
	Dual AD therapy	1	10.0
	AD + Antipsychotics	1	10.0

Absolute and relative frequencies of antidepressant (AD) classes and pharmacological augmentation strategies are reported. Percentages refer to patients receiving AD treatment (*N* = 10). Four different AD classes are separated: selective serotonin reuptake inhibitor (SSRI), selective noradrenalin reuptake inhibitor (SNRI), tricyclic ADs, and atypical ADs. Augmentation strategies include the use of two different AD classes (*dual AD therapy*), and the combination of an AD with an antipsychotic drug (*AD + Antipsychotics*)

### Study design

The study was conducted in a designated therapy room at the Clinic for Psychiatry and Psychotherapy (LMU University Hospital, Munich). Both dyad members, patient and therapist, wore a research-grade wrist-worn biosensor (*E4*, Empatica, Milan) on the non-dominant hand to minimize motion artifacts. Participants were seated in chairs at approximately a 60-degree angle relative to each other. A stationary video camera recorded the full course of each CBT session. Spoken content and speech were not recorded or analyzed. The wristbands continuously recorded physiological parameters referenced to the UNIX time system, allowing time synchronization across devices. Therapists were instructed to set a timestamp on their wristband at the beginning and end of each CBT session, which emitted a visible light signal captured in the video recording. The timestamps enabled temporal alignment of wearable data with video data. The dyad was left alone in the room during the CBT session. Session durations averaged M_duration_= 47.5 min (SD = 4.2 min), based on all recorded sessions (N_sessions_ = 24) within the follow-up sample (N_dyads_ = 12). After each CBT session, both patients and therapists completed standardized post-session reports. Patients also provided ratings of somatic and psychological symptom burden. The follow-up session followed the same setup.

### Materials

#### Movement data

The CBT sessions were recorded using a standard video camera with a frame rate of 25 frames per second (fps). The entire session, excluding the initial two minutes, was used for assessing movement IPS, using the software *Motion Energy Analysis V3.10* (MEA) [51]. MEA is a frame-differencing method quantifying grayscale pixel changes in pre-defined regions of interest (ROI) (for more details, see [51]). In the current study, two distinct ROIs were defined, focusing on head and upper body movement.

#### Heart rate

Empatica’s *E4* (Empatica, Milan) wristband sensor was used to continuously collect physiological data from both dyad members throughout the CBT sessions. During recording, data were streamed via Bluetooth to the *E4 Realtime* app and temporarily stored in the app’s cloud system. Following each CBT session, recorded data were transferred to the *E4 Connect* cloud platform, from which all raw datasets were subsequently downloaded and deleted from the platform. The wristband’s PPG sensor uses light signals at a sampling frequency of 64 Hz. Those light signals enable the extraction of cardiovascular dynamics, including HR, through pulse wave detection. For details on the preprocessing of PPG signals, see Sect.  3.2.1.

#### Self-report questionnaires

After each CBT session, both patient and therapist completed the *Berner Post-Session Report 2000* (*BPSR-P 2000/ BPSR-T 2000*) [52]. The BPSR-P comprises 22 items across eight subscales, whereas the BPSR-T consists of 27 items covering eleven dimensions. All items are rated on a seven-point bipolar Likert scale. In line with our research question, we selected alliance related items from both patient and therapist. Although Flückiger et al. [52] originally conceptualized the TA as a single dimension composed of three items in both questionnaires, we differentiated this construct for analytical clarity. Specifically, we separated the three TA items into a *state alliance* and a *trait alliance* dimension. This decision was theoretically motivated by the distinct temporal focus of the items: while one item targets the session-specific, momentary perception of the therapeutic bond (*state alliance*), the other two reflect a more generalized evaluation of the therapeutic relationship (*trait alliance*). For a detailed description of all selected dimensions with the belonging items, see Table 2 in the Supplementary Material. Patients’ depressive symptomatology was assessed using the German version of the *Beck Depression Inventory*—*Second Edition* (BDI-II) [53]. The BDI-II consists of 21 questions referring to the last 14 days, where each answer is scored with a value of 0 to 3. Higher total sum scores (BDI) indicate a more severe depressive symptomatology. Patients’ overall symptom severity was evaluated using the German version of the *Brief Symptom Inventory* (BSI) [54]. The BSI measures psychological distress and clinically relevant somatic symptoms experienced over the past seven days on a four-point scale. The questionnaire consists of 53 items covering the following nine dimensions: somatization, obsession-compulsion, interpersonal sensitivity, depression, anxiety, hostility, phobic anxiety, paranoid ideation, and psychoticism. *Global Severity Index* (GSI) as a key metric from the BSI reflects on client’s overall psychological distress experience. In our follow-up sample, BDI and GSI scores were assessed after both sessions. For analyzing therapy outcome, we were particularly interested in the change of GSI (∆GSI) and BDI (∆BDI) over time. Positive values of both difference scores indicate a reduction of patients’ symptom severity over time, while negative values indicate an increase. For descriptive statistics, see Tables 3, 4, 5 and 6 in the Supplementary Material.

### Data analysis

Data pre-processing and analysis of interpersonal physiological and movement synchrony were conducted using *R V4.4.0* [55] in *RStudio 2024.09.01.394* [56] and *Python 3.13.3* [57] in *Visual Studio Code*.

### Movement synchrony

#### Preprocessing and synchrony calculation

To account for the non-stationarity of movement data and the potential temporal delays between dyadic responses, a lagged windowed cross-correlation analysis was employed for each dyad and both ROIs, as extracted from MEA. The analysis was conducted using the *rMEA* package [58]. Raw MEA time series, sampled at 25 Hz, were initially imported. To improve signal quality, potential movement outliers were removed using the rMEA::MEAoutlier function. Outliers were defined as data points exceeding a dynamic threshold of ten times the standard deviation, applied in the positive direction. This way, only unusually high movement values were considered as outliers and thus excluded from further analyses. Following outlier correction, all timeseries were standardized using rMEA::MEAscale to ensure comparability across dyads and sessions. Subsequently, lagged cross-correlations were computed using the rMEA::MEAccf function. The cross-correlation function (CCF) was calculated over rolling windows of 60 s with 30-s increments, using a symmetric lag range of ± 5 s. This allowed the detection of delayed but temporally aligned patterns of movement between interactants within a specific time range. CCF values were calculated with non-absolute values, preserving the directionality of synchrony, and allowing us to differentiate in-phase synchrony from anti-phase synchrony. To enable statistical comparability, all CCF coefficients were Fisher z-transformed. To derive movement synchrony indices from the CCF matrices, a peak-picking algorithm was applied within each window. Two types of synchrony were computed per window: in-phase synchrony, based on all positive CCF values, and anti-phase synchrony, based on negative CCF values. For anti-phase synchrony, the strongest negative CCF value per window was identified by the maximum of the absolute negative CCF values. The negative sign was then reintroduced to preserve the directionality. For each dyad, peak values were aggregated across all analysis windows by calculating the mean for both synchrony types (i.e., in- and anti-phase), separately for both ROIs (i.e., head and body movement). This procedure yielded four movement based IPS metrics per dyad: in-phase head synchrony, anti-phase head synchrony, in-phase body synchrony, and anti-phase body synchrony. For descriptive statistics of those synchrony indices see Table 7 in the Supplementary Material.

### Control analysis

To ensure that the computed IPS indices capture genuine interpersonal movement coordination rather than statistical artifacts, a pseudosynchrony control analysis was conducted. Two surrogate data approaches were applied, described by Moulder et al. [59]: *data shuffling* and *section sliding.* These procedures were applied to the movement data from the initial session of the follow-up group. Following the analytical rationale of Plank et al. [60], based on Moulder et al. [59], we assessed (i) whether movement synchrony showed temporal structure across the session, and (ii) whether significant alignment could be observed within short time windows of the entire CBT session (i.e., 10-s segments). Pseudosynchrony metrics for both control approaches were computed using the same methodology and parameter settings as applied for real movement IPS calculation (i.e., lagged windowed cross-correlation and peak-picking). For each of the four IPS movement indices (i.e., in- and anti-phase IPS for head and body movements), Bayesian one-sample t tests were performed to compare real synchrony scores against those pseudosynchrony scores, each based on 200 surrogate iterations per dyad. The results revealed strong to extreme evidence for genuine synchrony in all four real-dyad IPS movement indices. Paired t-tests further confirmed these effects (for all *p* < 0.001). Those results support both hypotheses (i) and (ii) (for details, see Table 8 in the Supplementary Material), validating the applied lagged cross-correlation approach as a robust and sensitive method for quantifying IPS based on MEA-derived motion energy in this sample.

### Heart rate synchrony

#### Preprocessing

PPG time series from each participant were pre-processed using a protocol adapted from Campanella et al. [61]: First, raw PPG signals sampled at 64 Hz were segmented into 60-second windows with 15-second overlaps, corresponding to a final HR sampling frequency of 1/15 Hz. Each window was band-pass filtered using a Chebyshev type II filter (0.5–5 Hz, order = 4, stopband attenuation = 20dB) [61–62]. Peaks in the filtered signal were identified with a minimum inter-peak distance of 0.4 s. Inter-beat intervals (IBI) were computed, with a constraint of IBIs to lie between 0.4 and 1.2 s. This constraint refers to the assumption of HR to be within the physiologically plausible range of 50 beats per minute (bpm) to 150 bpm, especially when considering the setting of participants with no heart disease and a measurement while staying seated. IBI signals were then converted to a HR signal. Subsequently, the resulting HR time series was cleaned using a relative deviation filter: if the percentage change between two consecutive HR values exceeded 25%, the latter value was marked as incorrect [63]. If fewer than 25% of values in the whole HR time series were missing, linear interpolation was applied. To ensure the temporal plausibility of the HR signal, a second custom quality check was applied. For each HR time series, the relative change between each value and its counterpart two minutes prior was computed. If the deviation exceeded 25%, the value was flagged as physiologically implausible. Dyads in which either the patient’s or therapist’s pre-processed HR time series showed more than 25% of missing or corrected values were excluded from analysis. This resulted, as mentioned earlier, in the exclusion of three dyads (see Sect.  2.1).

#### Synchrony calculation

Similar to movement IPS calculation (see Sect.  3.1.1), HR synchrony was calculated using lagged windowed cross-correlation. HR time series were first normalized across sessions and dyads by using the rMEA::MEAscale function. Cross-correlation matrices were computed using rMEA: MEAccf across 5-minute windows with 2-minute increments, and ± 30-second lag range. Non-absolute CCF values were used for HR synchrony to distinguish the directionality of synchrony (i.e., in-phase versus anti-phase). All CCF coefficients were Fisher z-transformed for comparability. The peak-picking algorithm was applied to each CCF window. Afterward, all peaks per dyad were aggregated, resulting in two HR synchrony indices per dyad: in-phase and anti-phase HR synchrony (for details about the peak-picking method, see Sect.  3.1.1). For descriptive statistics of those synchrony indices see Table 7 in the Supplementary Material.

#### Control analysis

To ensure that calculated HR synchrony reflected genuine interpersonal physiological coupling, a pseudosynchrony control analysis was performed using the same surrogate data procedures as described in Sect.  2.4.1.2 (i.e., dyad-wise *data shuffling* and *section sliding*) [59]. Both approaches were applied to HR data from the initial session of the follow-up group. All surrogate datasets were processed using the same lagged windowed cross-correlation and peak-picking procedure as for real HR synchrony analysis (i.e., 5-min windows, 2-min increments, ± 30-s lag). To evaluate (i) the temporal structure of HR synchrony, and (ii) synchrony within short-term segments (here defined as 1-minute intervals), Bayesian one-sample t-tests were performed comparing real dyad synchrony against pseudosynchrony distributions (n_permutation_= 200). The results revealed strong to extreme evidence for genuine synchrony in both real in-phase and anti-phase components across dyad shuffling (BF_10_ = 37.9 and 29.7, respectively) and segment shuffling (BF_10_ = 2143.9 and 2510.7). Paired t-tests further confirmed these effects (for all *p* < 0.002). Together, these results support the robustness of the applied calculation method for IPS in HR dynamics.

#### Descriptive statistics

**Fig. 1:**
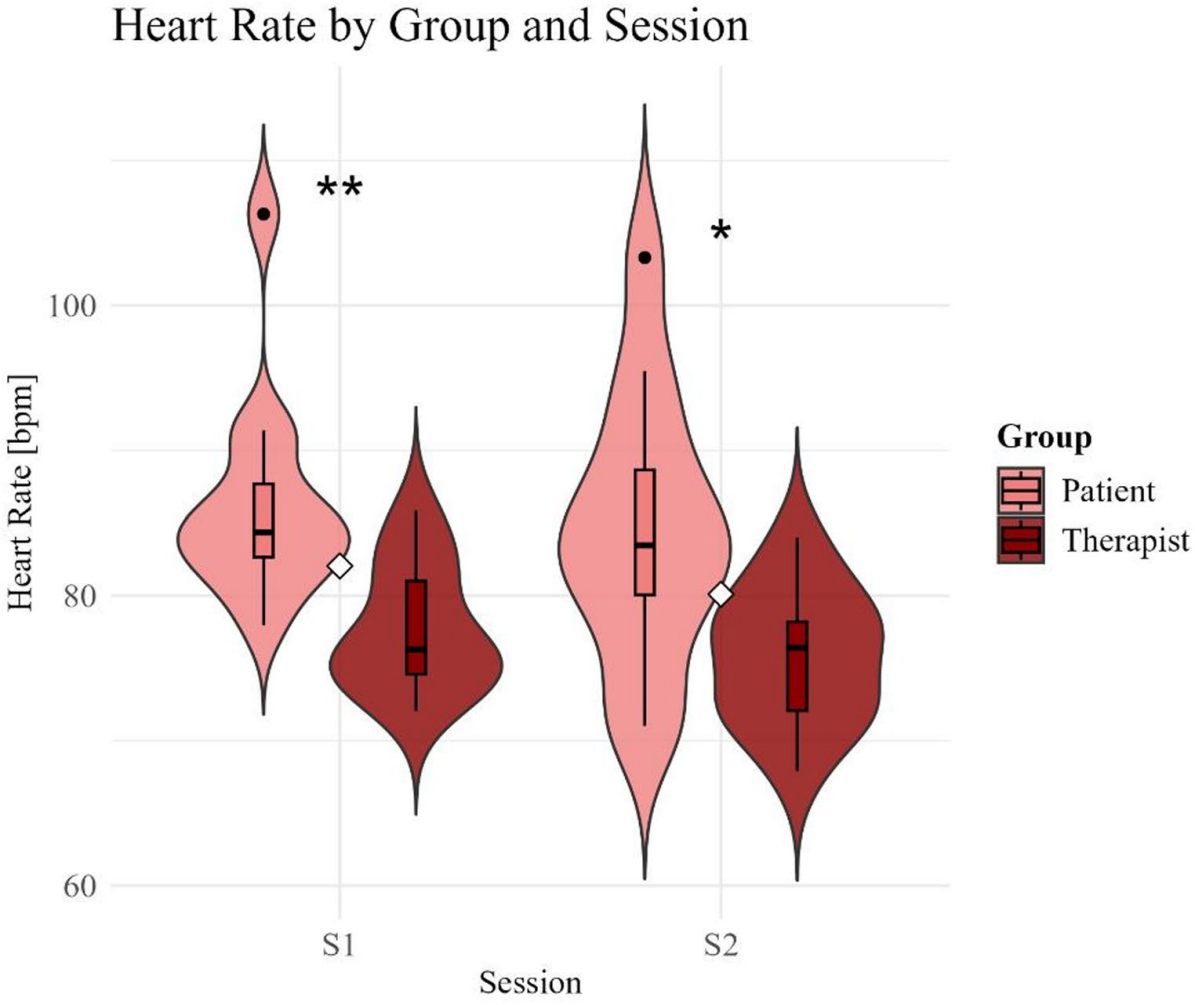
Comparison of heart rate means between patients and therapists. Violin plots with boxplots illustrate the distribution of mean heart rates, measured in beats per minute (bpm). Heart rates were measured in two sessions per dyad. Patients’ data are displayed in light red, while therapists’ data are shown in dark red. A paired Wilcoxon signed-rank test revealed significant differences in mean heart rates between patients and their belonging therapists in both sessions (Session 1 (S1): *p* = 0.002**; Session 2 (S2): *p* = 0.034*). Patients exhibited significantly higher heart rate values compared to therapists. *p < 0.05, **p < 0.01

A paired Wilcoxon signed-rank test revealed significant differences in mean HR between patients and their respective therapists across both CBT sessions. In both sessions, patients exhibited significantly higher mean HR values than their therapists. In the first recorded session (S1) patients (P) had a mean HR_P_S1_ = 86.4 bpm (SD = 7.4) compared to therapists’ (T) mean HR_T_S1_ = 77.7 bpm (SD = 4.3), *z* = 2.8, *W* = 75, *p* = 0.002. In the follow-up session (S2), the pattern remained consistent, with patients showing a mean HR_P_S2_ = 84.4 bpm (SD = 9.3) compared to therapists’ mean HR_T_S2_ = 75.8 bpm (SD = 4.7), *z* = 2.2, *W* = 66, *p* = 0.034. A summary of the HR statistics are presented in Table 4 and Fig. 1. Within the patient group, no significant differences in mean HR values were observed for the categorial variables gender, smoking status, alcohol use, or number of psychiatric diagnoses (all *p* > 0.05, see Supplementary Material, Table 9). Exploratory subgroup analyses further examined whether dyad gender composition was associated with differences in HR and synchrony measures. Patients in mixed-sex dyads (*N* = 6) showed significantly higher mean HR values (HR = 90.5 bpm) compared to patients in same-sex dyads (HR = 82.4 bpm), *U* = 5, *p* = 0.041. Additionally, mixed-sex dyads showed significantly higher ∆GSI values (*U* = 3, *p* = 0.015), and higher HR antiphase synchrony scores (*U* = 5, *p* = 0.041). For a summary of all variables see Table 10 in the Supplementary Material. However, given the small sample and subgroup sizes no adjustment for potential confounding variables was feasible and these findings should be interpreted with caution.

**Table 4:** Descriptives of heart rate measurements

Group	Session 1			Session 2		
	*n*	M [bpm]	SD	*n*	M [bpm]	SD
*Heart rate*						
Patients	12	86.4	7.4	12	84.4	9.3
Therapists	12	77.7	4.3	12	75.8	4.7

Mean (*M*) and standard deviation (*SD*) of heart rate measurements in each group are reported for both sessions of the follow-up sample. The heart rate was measured with a wristband sensor. Mean values are reported in beats per minute (bpm)

### Statistical analysis

Given that both HR and movement IPS scores significantly exceeded surrogate synchrony values, both modalities were considered in the following analyses to (1) investigate the relationship between objective synchrony measures and subjective alliance ratings, and (2) examine whether IPS or subjective ratings are related to short-term therapy outcome.

For the first part (1), correlation analyses were conducted using data from the initial session of the follow-up group: patients’ and therapists’ post-session reports, and HR and movement synchrony indices.

For the second part (2), two outcome variables were included in the correlation analysis: ΔGSI, and ΔBDI. Variables that were significantly correlated with either one of the outcome variables were subsequently entered as predictors into a simple linear regression model. All linear regression models met the necessary statistical requirements, including the independence of residuals and homoscedasticity.

## Results

### Interpersonal synchrony and post-session ratings

In-phase HR synchrony was positively associated with therapists’ *state alliance* ratings (*ρ* = 0.651, *p* = 0.022). Notably, none of the patients’ alliance ratings showed a significant association with any IPS variable. For a detailed summary of all correlations, see Tables 11, 12, 13, 14 and 15 in the Supplementary Material.

### Prediction of symptom change

**Fig. 2:**
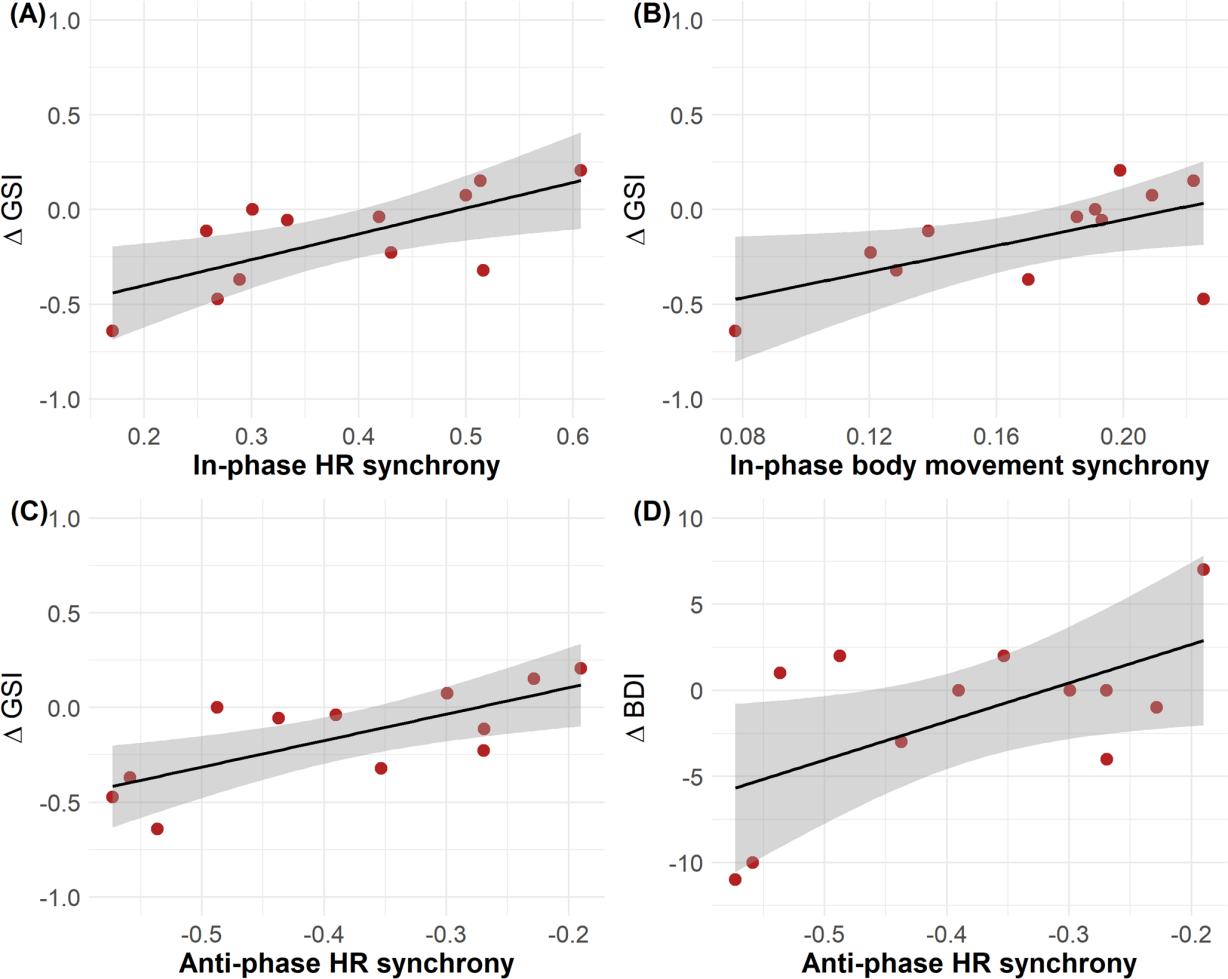
Interpersonal synchrony as a predictor for symptom change. Changes in patients’ Global Severity Index (ΔGSI) and depressive symptom severity (ΔBDI) were significantly predicted by interpersonal synchrony indices. Positive ΔGSI and ΔBDI values indicate a reduction from patients’ initial GSI/BDI scores over time, while negative values indicate an increase in symptomatology. ΔGSI and ΔBDI values of zero reflect no change in patients’ symptom ratings over time. A linear trendline and its 95% confidence interval (shaded area) illustrate the prediction of both change variables. **A**, **B** Higher in-phase synchrony for heart rate (HR) and movement during the initial CBT session were linked to a decrease in patients’ GSI over time, while lower in-phase synchrony indices were linked to an increase in symptomatology. **C**, **D** Greater anti-phase HR synchrony during the initial CBT session was linked to an increase in patients’ GSI and BDI scores over time, while lower anti-phase synchrony scores were linked to a decrease in symptomatology. **A** In-phase HR synchrony predicted ΔGSI, explaining 48% of the variance. **B** In-phase body synchrony predicted ΔGSI, explaining 37% of its variance. **C** Anti-phase HR synchrony predicted ΔGSI, explaining 52% of its variance. **D** Anti-phase HR synchrony predicted ΔBDI, explaining 36% of its variance

Simple linear regression models confirmed that three IPS variables significantly predicted ∆GSI: in-phase HR synchrony (*R*^2^ = 0.483, *F*(1,10) = 9.356, b = 1.359, *p* = 0.012), anti-phase HR synchrony (*R*^2^ = 0.524, *F*(1,10) = 10.997, b = 1.396, *p* = 0.008), and in-phase body synchrony (*R*^2^ = 0.367, *F*(1,10) = 5.801, b = 3.437, *p* = 0.037). In addition, anti-phase HR synchrony significantly predicted ΔBDI (*R*^2^ = 0.355, *F*(1,10) = 5.493, b = 22.350, *p* = 0.041). As shown in Fig. 2, higher in-phase HR and body synchrony were associated with a greater reduction in psychological distress experience, while low synchrony scores were related to symptom increase. Conversely, higher anti-phase HR synchrony was associated with an increase in both GSI and BDI over time.

Among the subjective post-session ratings, only therapists’ assessments of *state alliance* were significantly associated with both ΔGSI (*ρ* = 0.609, *p* = 0.035) and ΔBDI (*ρ* = 0.626, *p* = 0.029). However, when included in a linear regression model, therapists’ ratings significantly predicted only ΔBDI (*R*^2^ = 0.407, *F*(1,11) = 6.859, b = 2.857, *p* = 0.026), not ΔGSI (*p* > 0.05). An overview of the correlation matrices is provided in Tables 16, 17 and 18 (see Supplementary Material), and a summary of all linear regression models is presented in Table 5.

**Table 5:** Models for predicting therapy outcome

Model 1	Predictor	Outcome																
	HR_sync_in	∆GSI																
Model 2	HR_sync_anti	∆GSI																
Model 3	HR_sync_anti	∆BDI																
Model 4	Body_sync_in	∆GSI																
Model 5	T_State_Alliance	∆GSI																
Model 6	T_State_Alliance	∆BDI																

	Model 1			Model 2			Model 3			Model 4			Model 5			Model 6		
*Predictor*	*E*	*SE*	*p*	*E*	*SE*	*p*	*E*	*SE*	*p*	*E*	*SE*	*p*	*E*	*SE*	*p*	*E*	*SE*	*p*
(Intercept)	− 0.672	0.180	0.004**	0.384	0.170	0.047*	7.139	3.852	0.094	-0.74	0.253	0.015*	− 0.394	0.140	0.018*	− 7.131	2.480	0.017*
HR_sync_in	1.359	0.444	0.012*															
HR_sync_anti				1.396	0.421	0.008**												
HR_sync_anti							22.350	9.536	0.041*									
Body_sync_in										3.437	1.427	0.037*						
T_State_Alliance													0.122	0.062	0.076			
T_State_Alliance																2.857	1.091	0.026*
Observations	12			12			12			12			12			12		
R^2^	0.483			0.524			0.355			0.367			0.281			0.407		
R^2^ adjusted	0.432			0.476			0.290			0.304			0.209			0.348		
F-statistic	9.356		0.012*	11.0		0.008**	5.493		0.041*	5.801		0.037*	3.914		0.076	6.859		0.026*

Six linear regression models were calculated to predict therapy outcome variables: change in patients’ Global Severity Index (∆GSI), and depressive symptom severity (∆BDI). Predictor variables were selected based on prior significant correlations. Three objective parameters were included: in-phase and anti-phase heart rate synchrony (HR_sync_in, HR_sync_anti), and in-phase body movement synchrony (Body_sync_in). Among subjective variables, only the therapists’ rating of state alliance (T_State_Alliance) showed a significant correlation and was therefore included. Notably, all models with objective predictor variables and Model 6 reached statistical significance, while in Model 5, the subjective predictor failed to reach significance. Estimate (*E*), standard error (*SE*), and p value (*p*) are reported. All significant p-values are reported in bold and *p < 0.05, **p < 0.01

## Discussion

Conventional subjective assessment of TA overlooks the intricate embodied intertwining of client and therapist through their physiological and motion. In the current transdiagnostic pilot study, we aimed to address this gap by incorporating motor and physiological data alongside subjective reports from therapists and patients in a naturalistic CBT follow-up design. Wristband sensors continuously measured heart rate dynamics, while questionnaires were used to capture subjective ratings of therapeutic alliance and patients’ symptomatology.

In-phase HR synchrony correlated positively with therapists’ alliance ratings, aligning with prior findings that physiological synchrony relates to the concept of TA [35]. In contrast, we did not find a significant correlation between patients’ alliance ratings and IPS indices. This discrepancy underscores the occurrence of divergent subjective experiences among dyad members.

In-phase HR synchrony explained 48%, while anti-phase HR synchrony explained 52% of the variance in ΔGSI. Specifically, higher in-phase HR synchrony indices were associated with a greater reduction in symptom intensity, whereas high anti-phase and low in-phase HR synchrony indices were linked to an increase in symptom intensity levels over time. In contrast, among subjective measures, only therapists’ state alliance ratings significantly predicted symptom change, specifically the change in depressive symptom severity. Further research is necessary to analyze the causal relationship, including whether specific psychiatric symptoms are associated with decreased HR synchrony in interaction in general, and whether the level of HR synchrony increases in parallel with decreasing symptom burden over time.

While wristbands showed potential for assessing interpersonal physiological coupling, results remain preliminary given the small, artifact-filtered subsample. As we were interested in physiological IPS, low data quality from even one dyad member led to the exclusion of the whole dyad. Wearable sensors are usually more sensitive to motion artifacts, leading to lower data quality. While most physiological IPS research traditionally relied on classical devices like electrocardiograms for HR measurements, our study investigated the potential of wrist-worn devices in capturing physiological dynamics. Those devices are less intrusive, which is important when aiming to capture naturalistic interaction, particularly in the setting of psychotherapy, where content is often quite intimate and distressing.

Additionally, the heterogeneity of our patient group might limit the generalizability of our findings. Nevertheless, among the participants in our study, over half presented with at least one diagnosis falling within the spectrum of affective disorders (ICD-10 F3 diagnosis) [50]. This cohort is notably representative of patients commonly encountered in the setting of CBT. Given the frequent occurrence of comorbid diagnoses among psychiatric patients in clinical practice, our findings underscore the potential utility of physiological IPS as a transdiagnostic measure for therapy outcomes. Based on the outcome of this explanatory study and previous analyses [33, 64, 65], there is converging evidence of the potential of portable biosensors in psychotherapy research, warranting future validation studies in larger samples and preferably longitudinal designs. Although our follow-up assessment period was relatively short, this timeframe was a pragmatic compromise, balancing the need for meaningful outcome assessment with the high patient turnover in a psychiatric clinic. Furthermore, due to the recruitment in routine care, the number of therapy sessions completed prior to study participation could not be fully standardized. Most dyads were assessed during the early phase of short-term CBT, although some variability remained. Differences in therapy stage may influence interpersonal and physiological processes. Notably, prior work suggests that the predictive strength of the TA is not linearly related to time; rather, evidence suggests that alliance assessments taken early on or toward the end of treatment appear to offer more insight into treatment efficacy compared to ratings made during therapy [16]. These findings support the idea that meaningful therapeutic change can indeed be captured within a relatively brief follow-up period. Nevertheless, despite the limited timeframe, the majority of our patients showed measurable changes in symptom severity. Due to the sample size, we could only run regression models with one predictor variable. Future studies with larger and diagnostically stratified samples, as well as extended follow-up periods, are needed to validate and extend these preliminary findings.

In conclusion, our study demonstrates the feasibility and transdiagnostic potential of applying wristband sensors to CBT sessions to capture interpersonal dynamics. In a low-threshold, naturalistic approach, HR synchrony emerged as a potential predictor for changes in patients’ symptom severity. Moreover, integrating the concept of embodiment into further research on evidence-based psychotherapy might have the potential to tailor therapy approaches and enhance patient-therapist matching.

## Supplementary Information

Below is the link to the electronic supplementary material.

Supplementary file1

## Data Availability

The participants of this study did not give written consent for their data to be shared publicly, so due to the sensitive nature of the research supporting data is not available.
