# Supplementary material for: Heart rate synchrony as an objective biomarker in psychotherapy: a wearable-based pilot study
Source: Eur Arch Psychiatry Clin Neurosci. 2026 May 19;276(5):2101–12. doi: 10.1007/s00406-026-02248-4 (PMC13481465; doi:10.1007/s00406-026-02248-4)
Supplement: Supplementary file 1 [file 406_2026_2248_MOESM1_ESM.docx]

**Supplementary Material**

Title: **Heart rate synchrony as an objective biomarker in psychotherapy: a wearable-based pilot study**
Journal: European Archives of Psychiatry and Clinical Neuroscience

Clara C. Gernert^1,2*^and Christine M. Falter-Wagner^1,2*^
^1^ Department of Psychiatry and Psychotherapy, LMU University Hospital, Munich, Germany

^2^ German Centre for Mental Health (Deutsches Zentrum für Psychische Gesundheit, DZPG), Site Munich-Augsburg, Germany

Corresponding author: clara.gernert@med.uni-muenchen.de

**Table 1** Comparison of descriptives between included and excluded dyads

|  |  | | **Group** | | |  |  |  |
| --- | --- | --- | --- | --- | --- | --- | --- | --- |
|  |  | | **Inclusion** | | **Exclusion** | ***X^2^*** | ***U*** | ***p*** |
| Patients' gender, n (%) | | Female | | 8 (66.7) | 9 (69.2) | 0.2 |  | .891 |
|  | | Male | | 4 (33.3) | 4 (30.8) |  |  |  |
| Therapists' gender, n (%) | | Female | | 7 (87.5) | 4 (44.4) | 3.4 |  | .064 |
|  | | Male | | 1 (12.5) | 5 (55.6) |  |  |  |
| Gender in dyad, n (%) | | Same-sex | | 6 (50.0) | 6 (46.2) | 0.0 |  | .848 |
|  | | Mixed-sex | | 6 (50.0) | 7 (53.8) |  |  |  |
| Psychiatric diagnoses, n (%) | | Single | | 8 (66.7) | 9 (69.2) | 0.0 |  | .891 |
|  | | Multiple | | 4 (33.3) | 4 (30.8) |  |  |  |
| Antidepressants, n (%) | | None | | 2 (16.7) | 6 (46.2) | 2.5 |  | .114 |
|  | | Positive | | 10 (83.3) | 7 (53.8) |  |  |  |
| Smoking status, n (%) | | Negative | | 9 (75.0) | 8 (61.5) | 0.5 |  | .471 |
|  | | Positive | | 3 (25.0) | 5 (38.5) |  |  |  |
| Alcohol consumption, n (%) | | Negative | | 8 (66.7) | 5 (38.5) | 2.0 |  | .158 |
|  | | Positive | | 4 (33.3) | 8 (61.5) |  |  |  |
| Age, mean [years] | | Patients | | 35.1 | 37.1 |  | 86.0 | .682 |
|  | | Therapists | | 28.9 | 32.4 |  | 24.5 | .285 |
| *Note*. Descriptives of included dyads (N = 12) were compared to excluded dyads (N = 13). Dyads without a follow-up session, with low data quality, or with technical failures were excluded from further analysis. Group differences for categorical variables were examined using chi-square tests and Mann-Whitney U tests for continuous variables (i.e., age). Psychiatric diagnoses refer to the number of current diagnoses. Antidepressant medication and smoking status refer to the current status at the time of assessment. Alcohol consumption refers to moderate regular or social use, patients with acute substance abuse were not included. No statistically significant differences were observed between included and excluded dyads (for all *p* > .05). | | | | | | | | |

**Table 2** Overview of selected items of post-session reports

|  | *Group* | *BPSR-Dimension* | *Abbreviation* | *German Items* [Number in the BPSR]* | *Translated Items* |  |
| --- | --- | --- | --- | --- | --- | --- |
|  | Patient | State Alliance | P_State_Alliance | Heute habe ich mich in der Beziehung zum Therapeuten wohlgefühlt. [1] | Today I felt comfortable in the relationship with my therapist. | |
|  |  | Trait Alliance | P_Trait_Alliance | Der Therapeut und ich verstehen einander. [7] | The therapist and I understand each other. | |
|  |  |  |  | Ich glaube der Therapeut ist wirklich an meinem Wohlergehen interessiert. [9] | I believe the therapist is genuinely interested in my well-being. | |
| Therapist | | State Alliance | T_State_Alliance | Heute habe ich mich in der Beziehung zum Patienten wohlgefühlt. [1] | Today I felt comfortable in the relationship with my patient. | |
|  | | Trait Alliance | T_Trait_Alliance | Der Patient und ich verstehen einander. [2] | The patient and I understand each other. | |
|  | |  |  | Der Patient und ich arbeiten an gemeinsamen Zielen. [3] | The patient and I are working toward shared goals. | |

*Note.* The Berner Post-Session Report (*BPSR*; Flückiger et al., 2010) is a multidimensional questionnaire designed to assess psychotherapy process and the therapeutic relationship from both patient and therapist perspectives. Patients’ questionnaire (*BPSR-P*) encompasses 22 items, while therapists (*BPSR-T*) give ratings on 27 items. We selected two dimensions for both, patients and therapists, focusing on alliance-related variables. The table gives an overview of the selected dimensions for both groups, patients and therapists, its original German wording, the corresponding item number within the BPSR, and an English translation.

**Table 3** Descriptive statistics of alliance ratings

|  |  | **Session 1** | | | |  | **Session 2** | | | |
| --- | --- | --- | --- | --- | --- | --- | --- | --- | --- | --- |
| *Group* | *Dimension* | *n* | *M* | *SD* | *Shapiro-Wilk*  *[p-value]* |  | *n* | *M* | *SD* | *Shapiro-Wilk [p-value]* |
| Patient | State Alliance | 12 | 1.833 | 1.467 | 0.775 [0.005] |  | 12 | 2.167 | 1.193 | 0.725 [0.001] |
|  | Trait Alliance | 12 | 4.00 | 2.296 | 0.793 [0.008] |  | 12 | 4.750 | 1.603 | 0.794 [0.008] |
| Therapist | State Alliance | 12 | 2.000 | 1.128 | 0.754 [0.003] |  | 12 | 2.083 | 0.669 | 0.809 [0.012] |
|  | Trait Alliance | 12 | 3.750 | 1.215 | 0.940 [0.495] |  | 12 | 2.167 | 1.193 | 0.725 [0.001] |

*Note.* Mean (*M*) and standard deviation (*SD*) are reported for each of the selected alliance ratings. All dimensions were tested for normality using the Shapiro-Wilk test.

**Table 4** Descriptive statistics of patients’ depressive symptom severity

| **BDI score** | *Depression Severity* | **Session 1** | | **Session 2** | |
| --- | --- | --- | --- | --- | --- |
|  |  | *n* | *%* | *n* | *%* |
| [0; 13] | None | 3 | 25.0 | 2 | 16.7 |
| [14; 19] | Mild | 3 | 25.0 | 3 | 25.0 |
| [20; 28] | Moderate | 1 | 8.3 | 2 | 16.7 |
| [29; 63] | Severe | 5 | 41.7 | 5 | 41.7 |
|  | Total | 12 | 100 | 12 | 100 |

*Note.* Absolute (*n*) and relative (*%*) frequencies of patients’ rating scores about their depressive symptomatology are reported. Depressive symptomatology was assessed using the questionnaire BDI-II (Hautzinger et al., 2009). Higher BDI scores indicate greater severity of depressive symptomatology. Subgroups were built by established cut-off scores.

**Table 5** Descriptive statistics of patients’ Global Severity Index (GSI)

| **GSI Score** | **Session 1** | | | | **Session 2** | | | |
| --- | --- | --- | --- | --- | --- | --- | --- | --- |
|  | *n* | *%* | *M* | *SD* | *n* | *%* | *M* | *SD* |
| [0; 1[ | 2 | 16.7 | 0.6 | 0.2 | 1 | 8.3 | 0.4 | 0 |
| [1; 2[ | 8 | 66.7 | 1.5 | 0.2 | 8 | 66.7 | 1.5 | 0.2 |
| [2; 3[ | 1 | 8.3 | 2.4 | 0 | 2 | 16.7 | 2.4 | 0.2 |
| [3; 4[ | 1 | 8.3 | 3.1 | 0 | 1 | 8.3 | 3.4 | 0 |
| Total | 12 | 100.0 | 1.6 | 0.7 | 12.0 | 100.0 | 1.7 | 0.7 |

*Note.* Absolute (*n*) and relative frequencies (*%*) of patients’ rating scores about their Global Severity Index (GSI), separated session-wise for the follow-up group. Mean scores (*M*) with standard deviation (*SD*) were calculated separately. The maximum GSI value is 4. Overall, higher values indicate a higher symptom burden.

**Table 6** Descriptive statistics of therapy outcome variables

|  | **ΔScore** | | |  |
| --- | --- | --- | --- | --- |
|  | **< 0** | **0** | **> 0** |  |
|  | *n (%)* | *n (%)* | *n (%)* | *Total (%)* |
| **ΔGSI** | 8 (66.7) | 1 (8.3) | 3 (25.0) | 12 (100) |
| **ΔBDI** | 5 (41.7) | 3 (25.0) | 4 (33.3) | 12 (100) |

*Note.* Frequency distributions of the outcome variables change in the Global Severity Index (ΔGSI) and depressive symptom burden (ΔBDI). Negative values indicate an increase in symptom severity over time, while positive values reflect a reduction. A value of zero denotes no change between the two measured sessions for a given dyad.

**Table 7** Descriptive statistics of interpersonal synchrony scores

| **Synchrony Scores** | **Session 1** | | | |  | **Session 2** | | | | |  |
| --- | --- | --- | --- | --- | --- | --- | --- | --- | --- | --- | --- |
|  | *n* | *M* | *SD* | *Shapiro-Wilk  [p-value]* | |  | *n* | *M* | *SD* | *Shapiro-Wilk [p-value]* | |
| HR in-phase | 12 | 0.384 | 0.133 | 0.954 [0.703] | |  | 12 | 0.268 | 0.088 | 0.914 [0.238] | |
| HR anti-phase | 12 | -0.383 | 0.135 | 0.931 [0.391] | |  | 12 | -0.422 | 0.113 | 0.924 [0.319] | |
| Body in-phase | 12 | 0.172 | 0.046 | 0.919 [0.277] | |  | 12 | 0.175 | 0.034 | 0.962 [0.806] | |
| Body anti-phase | 12 | -0.107 | 0.037 | 0.792 [0.008] | |  | 12 | -0.112 | 0.027 | 0.957 [0.738] | |
| Head in-phase | 12 | 0.187 | 0.035 | 0.927 [0.347] | |  | 12 | 0.179 | 0.025 | 0.939 [0.483] | |
| Head anti-phase | 12 | -0.108 | 0.016 | 0.967 [0.883] | |  | 12 | -0.110 | 0.013 | 0.986 [0.998] | |

*Note.* Interpersonal synchrony was calculated for body and head movements, as well as heart rate. In-phase and anti-phase synchrony were analyzed separately. Mean (*M*) and standard deviation (*SD*) values are reported for each modality, across both sessions within the follow-up setting. Normality of the data was tested using the Shapiro-Wilk test.

**Table 8** Control analysis: comparison of surrogates’ versus real-dyads’ synchrony scores

|  |  |  | Bayesian t-test |  | Paired t-tests | |
| --- | --- | --- | --- | --- | --- | --- |
| *Modality* | *Surrogate method* | *Synchrony type* | *BF_10_* |  | *t* | *p-value* |
| **Body** | Data shuffling | In-phase | 1929.3 |  | 7.544 | < .001*** |
|  |  | Anti-phase | 270.4 |  | -5.870 | < .001*** |
|  | Section sliding | In-phase | 145100.1 |  | 12.230 | < .001*** |
|  |  | Anti-phase | 16525.8 |  | -9.697 | < .001*** |
| **Head** | Data shuffling | In-phase | 56643.1 |  | 11.124 | < .001*** |
|  |  | Anti-phase | 281226.4 |  | -13.234 | < .001*** |
|  | Section sliding | In-phase | 5400015.6 |  | 18.062 | < .001*** |
|  |  | Anti-phase | 50986409.8 |  | -22.763 | < .001*** |
| **HR** | Data shuffling | In-phase | 37.9 |  | 4.397 | .001** |
|  |  | Anti-phase | 29.70 |  | -4.226 | .001** |
|  | Section sliding | In-phase | 2143.9 |  | 7.641 | < .001*** |
|  |  | Anti-phase | 2510.7 |  | -7.788 | < .001*** |

*Note*. A control analysis was conducted for all three modalities, head and body movement, and heart rate (HR), using two surrogate data generation methods: *data shuffling* and *section sliding* (Moulder et al., 2018). For the *section sliding* method, segment size was set to 10-minutes for body and head movement, and 1-minute for HR. Synchrony and pseudosynchrony scores were computed using the lagged-windowed cross-correlation algorithm with non-absolute values, combined with the peak picking method. A Bayesian one-sample t-test indicated strong to extreme evidence for genuine synchrony for all variables and across both surrogate methods. These findings were further supported by a paired t-test. ** indicate p <.01, *** indicates *p* < .001.

**Table 9** Group differences in patients’ mean heart rate

| **Group** | **Category** |  |  | **HR** | |  | |
| --- | --- | --- | --- | --- | --- | --- | --- |
|  |  |  | *n* | *M [bpm]* | *SD* | *U* | *p* |
| Patients | Gender | Female | 8 | 84.6 | 4.8 | 21.0 | .461 |
|  |  | Male | 4 | 90.2 | 10.8 |  |  |
|  | Smoking status | Non smoker | 9 | 87.8 | 7.9 | 20.0 | .282 |
|  |  | Smoker | 3 | 82.4 | 4.2 |  |  |
|  | Alcohol use | None | 8 | 84.4 | 4.0 | 13.0 | .683 |
|  |  | Social / Regular | 4 | 90.5 | 11.3 |  |  |
|  | Psychiatric diagnoses | Single | 8 | 84.0 | 3.7 | 8.0 | .214 |
|  |  | Multiple | 4 | 91.3 | 10.9 |  |  |

*Note.* Patients’ mean (M) heart rate (HR) measured in beats per minute (bpm) are reported in four different subgroups. Group differences within each category were analysed using Mann-Whitney U tests. Psychiatric diagnoses refer to the number of current psychiatric diagnoses per patient. No statistically significant differences in HR were observed between patients’ subgroups (p > .05).

| **Table 10** Group differences between same-sex and mixed-sex dyads | | | | | | |  |
| --- | --- | --- | --- | --- | --- | --- | --- |
|  | | | Dyads | | Mann-Whitney U test | | |
|  |  | Same-sex (N = 6) | | Mixed-sex (N = 6) | *U* | *p* | |
| Heart rate, mean [bpm] | Patient | 82.4 | | 90.5 | 5 | .041* | |
|  | Therapist | 79.1 | | 76.3 | 24 | .394 | |
| Therapists' rating | Trait alliance | 3.7 | | 3.8 | 17.5 | 1.0 | |
|  | State alliance | 1.7 | | 2.3 | 14 | .542 | |
| Patients' rating | Trait alliance | 3.8 | | 4.2 | 15 | .681 | |
|  | State alliance | 1.8 | | 1.8 | 14 | .555 | |
| Patients' symptom burden | GSI | 1.6 | | 1.5 | 19 | .937 | |
|  | BDI | 26.2 | | 23.8 | 16 | .818 | |
| Symptom change | ∆GSI | -0.3 | | 0.0 | 3 | .015* | |
|  | ∆BDI | -2.7 | | -0.2 | 19 | .936 | |
| Synchrony indices | HR inphase | 0.3 | | 0.4 | 11 | .310 | |
|  | HR antiphase | -0.5 | | -0.3 | 5 | .041* | |
|  | Body inphase | 0.2 | | 0.2 | 9 | .180 | |
|  | Body antiphase | -0.1 | | -0.1 | 21 | .699 | |
|  | Head inphase | 0.2 | | 0.2 | 11 | .310 | |
|  | Head antiphase | -0.1 | | -0.1 | 15 | .699 | |

*Note.* HR = heart rate, GSI = global severity index, BDI = Beck Depression Inventory, ∆GSI = GSI change over time, ∆BDI = change in depressive symptoms over time. All values represent group means. Group differences between same-sex (N = 6) and mixed-sex dyads (N = 6) of the follow-up group (N = 12) were examined using Mann-Whitney U tests. Statistically significant differences were observed for patients’ mean HR, ∆GSI, and HR antiphase synchrony. However, given the small sample size and subgroup sizes, these findings should be interpreted with caution, as the statistical power is low and adjustment for potential confounding variables was not feasible.

* refers to p < .05.

**Table 11** Correlation matrix: interpersonal synchrony indices and patients’ baseline symptom severity

| Variables | *M* | *SD* | 1 | 2 | 3 | 4^ρ^ | 5 | 6 | 7 |
| --- | --- | --- | --- | --- | --- | --- | --- | --- | --- |
| 1. GSI_S1 | 1.556 | 0.711 |  |  |  |  |  |  |  |
| 2. BDI_S1 | 25.00 | 14.894 | **0.728**** |  |  |  |  |  |  |
|  |  |  | **[.265, .918]** |  |  |  |  |  |  |
| 3. Body_sync_in | 0.172 | 0.046 | -0.207 | -0.102 |  |  |  |  |  |
|  |  |  | [-.698, .416] | [-.639, .501] |  |  |  |  |  |
| 4. Body_sync_anti^ρ^ | -0.107 | 0.037 | 0.063 | -0.126 | 0.462 |  |  |  |  |
|  |  |  | [-.530, .615] | [-.653, .483] | [-.153, .819] |  |  |  |  |
| 5. Head_sync_in | 0.187 | 0.035 | -0.348 | -0.397 | **0.811**** | 0.336 |  |  |  |
|  |  |  | [-768, .282] | [-.791, .229] | **[.443, .945]** | [-.295, .763] |  |  |  |
| 6. Head_sync_anti | -0.108 | 0.016 | 0.438 | 0.332 | 0.549 | -0.007 | 0.397 |  |  |
|  |  |  | [-.182, .809] | [-.298, .761] | [-.037, .854] | [-.579, .569] | [-.229, .791] |  |  |
| 7. HR_sync_in | 0.384 | 0.133 | 0.491 | 0.553 | 0.372 | -0.203 | 0.054 | **0.693*** |  |
|  |  |  | [-.115, .831] | [-.030, .855] | [-.257, .779] | [-.696, .420] | [-.536, .609] | **[.198, .906]** |  |
| 8. HR_sync_anti | -0.383 | 0.135 | 0.412 | 0.189 | 0.096 | -0.315 | -0.120 | 0.424 | **0.764**** |
|  |  |  | [-.212, .797] | [-.431, .688] | [-.505, .635] | [-.753, .316] | [-.649, .488] | [-.198, .803] | **[.338, .930]** |

*Note*. *M* and *SD* represent mean and standard deviation. Pearson and Spearman (^ρ^) correlation coefficients were computed to examine the relationship between interpersonal synchrony and patients’ initial symptom burden, namely: patients’ global severity index (GSI_S1) and depressive symptom severity (BDI_S1), in-phase and anti-phase body movement synchrony (Body_sync_in, Body_sync_anti), in-phase and anti-phase head movement synchrony (Head_sync_in, Head_sync_anti), in-phase and anti-phase heart rate synchrony (HR_sync_in, HR_sync_anti). Values in square brackets indicate the 95% confidence interval for each correlation. * indicates p < .05. ** indicates p < .01.

**Table 12** Correlation matrix: patients’ alliance ratings and baseline symptom severity

| Variables | *M* | *SD* | 1 | 2 | 3^ρ^ |
| --- | --- | --- | --- | --- | --- |
| 1. GSI_S1 | 1.556 | 0.711 |  |  |  |
| 2. BDI_S1 | 25.000 | 14.894 | **0.728**** |  |  |
|  |  |  | **[.265, .918]** |  |  |
| 3. P_State_Alliance^ρ^ | 1.833 | 1.467 | -0.530 | -0.118 |  |
|  |  |  | [-.846, .063] | [-.648, .489] |  |
| 4. P_Trait_Alliance^ρ^ | 4.000 | 2.296 | **-0.729**** | -0.284 | **0.707*** |
|  |  |  | **[-.919, -.267]** | [-.738, .347] | **[.224, .911]** |

*Note*. *M* and *SD* represent mean and standard deviation. Pearson and Spearman (^ρ^) correlation coefficients were computed to examine the relationship between patients’ alliance ratings and patients’ initial symptom burden, namely: patients’ global severity index (GSI_S1) and depressive symptom severity (BDI_S1), and patients’ ratings of state alliance (P_State_Alliance) and trait alliance (P_Trait_Alliance). Values in square brackets indicate the 95% confidence interval for each correlation. * indicates p < .05. ** indicates p < .01. *** indicates p < .001.

**Table 13** Correlation matrix: therapists’ alliance ratings and patients’ baseline symptom severity

| Variable | *M* | *SD* | 1 | 2 | 3^ρ^ |
| --- | --- | --- | --- | --- | --- |
| 1. GSI_S1 | 1.556 | 0.711 |  |  |  |
| 2. BDI_S1 | 25.000 | 14.894 | **0.728**** |  |  |
|  |  |  | **[.265, .918]** |  |  |
| 3. T_State_Alliance^ρ^ | 2.000 | 1.128 | 0.175 | 0.103 |  |
|  |  |  | [-.443, .681] | [-.501, .639] |  |
| 4. T_Trait_Alliance | 3.750 | 1.215 | 0.146 | -0.010 | 0.188 |
|  |  |  | [-.467, .664] | [-.581, .567] | [-.432, .688] |

*Note*. *M* and *SD* represent mean and standard deviation. Pearson and Spearman (^ρ^) correlation coefficients were computed to examine the relationship between therapists’ post-session report ratings and patients’ initial symptom burden, namely: patients’ global severity index (GSI_S1) and depressive symptom severity (BDI_S1), and therapists’ ratings of state alliance (T_State_Alliance) and trait alliance (T_Trait_Alliance). Values in square brackets indicate the 95% confidence interval for each correlation. * indicates p < .05. ** indicates p < .01.

**Table 14** Correlation matrix: patients’ alliance ratings and synchrony indices

| Variables | *M* | *SD* | 1 | 2^ρ^ | 3 | 4 | 5 | 6 | 7^ρ^ |
| --- | --- | --- | --- | --- | --- | --- | --- | --- | --- |
| 1. Body_sync_in | 0.172 | 0.046 |  |  |  |  |  |  |  |
|  |  |  |  |  |  |  |  |  |  |
| 2. Body_sync_anti^ρ^ | -0.107 | 0.037 | 0.462 |  |  |  |  |  |  |
|  |  |  | [-.153, .819] |  |  |  |  |  |  |
| 3. Head_sync_in | 0.187 | 0.035 | **0.811**** | 0.336 |  |  |  |  |  |
|  |  |  | **[.443, .945]** | [-.295, .763] |  |  |  |  |  |
| 4. Head_sync_anti | -0.108 | 0.016 | 0.549 | -0.007 | 0.397 |  |  |  |  |
|  |  |  | [-.037, .854] | [-.579, .569] | [-.229, .791] |  |  |  |  |
| 5. HR_sync_in | 0.384 | 0.133 | 0.372 | -0.203 | 0.054 | **0.693*** |  |  |  |
|  |  |  | [-.257, .779] | [-.696, .420] | [-.536, .609] | **[.198, .906]** |  |  |  |
| 6. HR_sync_anti | -0.383 | 0.135 | 0.096 | -0.315 | -0.120 | 0.424 | **0.764**** |  |  |
|  |  |  | [-.505, .635] | [-.753, .316] | [-.649, .488] | [-.198, .803] | **[.338, .930]** |  |  |
| 7. P_State_Alliance^ρ^ | 1.833 | 1.467 | 0.405 | -0.214 | 0.420 | -0.162 | -0.015 | -0.258 |  |
|  |  |  | [-.220, .794] | [-.701, .411] | [-.203, .801] | [-.673, .454] | [-.584, .564] | [-.724, .371] |  |
| 8. P_Trait_Alliance^ρ^ | 4.000 | 2.296 | 0.259 | -0.681 | 0.194 | -0.194 | 0.097 | -0.075 | **0.707*** |
|  |  |  | [-.370, .725] | [-.681, .443] | [-.428, .691] | [-.691, .428] | [-.505, .636] | [-.622, .521] | **[.224, .911]** |

*Note*. *M* and *SD* represent mean and standard deviation. Pearson and Spearman (^ρ^) correlation coefficients were computed to examine the relationship between patients’ alliance ratings and interpersonal synchrony, namely: in-phase and anti-phase body movement synchrony (Body_sync_in, Body_sync_anti), in-phase and anti-phase head movement synchrony (Head_sync_in, Head_sync_anti), in-phase and anti-phase heart rate synchrony (HR_sync_in, HR_sync_anti), and patients’ ratings of state alliance (P_State_Alliance) and trait alliance (P_Trait_Alliance). Values in square brackets indicate the 95% confidence interval for each correlation. * indicates p < .05. ** indicates p < .01. *** indicates p < .001.

**Table 15** Correlation matrix: therapists’ alliance ratings and synchrony indices

| Variables | *M* | *SD* | 1 | 2^ρ^ | 3 | 4 | 5 | 6 | 7^ρ^ |
| --- | --- | --- | --- | --- | --- | --- | --- | --- | --- |
| 1. Body_sync_in | 0.172 | 0.046 |  |  |  |  |  |  |  |
|  |  |  |  |  |  |  |  |  |  |
| 2. Body_sync_anti^ρ^ | -0.107 | 0.037 | 0.462 |  |  |  |  |  |  |
|  |  |  | [-.153, .819] |  |  |  |  |  |  |
| 3. Head_sync_in | 0.187 | 0.035 | **0.811**** | 0.336 |  |  |  |  |  |
|  |  |  | **[.443, .945]** | [-.295, .763] |  |  |  |  |  |
| 4. Head_sync_anti | -0.108 | 0.016 | 0.549 | -0.007 | 0.397 |  |  |  |  |
|  |  |  | [-.037, .854] | [-.579, .569] | [-.229, .791] |  |  |  |  |
| 5. HR_sync_in | 0.384 | 0.133 | 0.372 | -0.203 | 0.054 | **0.693*** |  |  |  |
|  |  |  | [-.257, .779] | [-.696, .420] | [-.536, .609] | **[.198, .906]** |  |  |  |
| 6. HR_sync_anti | -0.383 | 0.135 | 0.096 | -0.315 | -0.120 | 0.424 | **0.764**** |  |  |
|  |  |  | [-.505, .635] | [-.753, .316] | [-.649, .488] | [-.198, .803] | **[.338, .930]** |  |  |
| 7. T_State_Alliance^ρ^ | 2.000 | 1.128 | 0.246 | 0.183 | 0.061 | 0.495 | **0.651*** | 0.438 |  |
|  |  |  | [-.382, .719] | [-.437, .685] | [-.532, .613] | [-.110, .832] | **[.124, .892]** | [-.181, .809] |  |
| 8. T_Trait_Alliance | 3.750 | 1.215 | -0,282 | 0.043 | -0.348 | -0.292 | -0.238 | 0.071 | 0.188 |
|  |  |  | [-.737, .348] | [-.544, .602] | [-.768, .282] | [-.742, .338] | [-.714, .389] | [-.524, .620] | [-.432, .688] |

*Note*. *M* and *SD* represent mean and standard deviation. Pearson and Spearman (^ρ^) correlation coefficients were computed to examine the relationship between therapists’ alliance ratings and interpersonal synchrony, namely: in-phase and anti-phase body movement synchrony (Body_sync_in, Body_sync_anti), in-phase and anti-phase head movement synchrony (Head_sync_in, Head_sync_anti), in-phase and anti-phase heart rate synchrony (HR_sync_in, HR_sync_anti), and therapists’ ratings of state alliance (T_State_Alliance) and trait alliance (T_Trait_Alliance). Values in square brackets indicate the 95% confidence interval for each correlation. * indicates p < .05. ** indicates p < .01.

**Table 16** Correlation matrix: patients’ alliance ratings and therapy outcome

| Variables | *M* | *SD* | 1 | 2 | 3^ρ^ |
| --- | --- | --- | --- | --- | --- |
| 1. ΔGSI | -0.150 | 0.260 |  |  |  |
| 2. ΔBDI | -1.417 | 5.054 | 0.482 |  |  |
|  |  |  | [-.127, .827] |  |  |
| 3. P_State_Alliance^ρ^ | 1.833 | 1.467 | 0.110 | 0.145 |  |
|  |  |  | [-.258, .779] | [-.468; .664] |  |
| 4. P_Trait_Alliance^ρ^ | 4.000 | 2.296 | 0.399 | 0.536 | **0.707*** |
|  |  |  | [-.227, .791] | [-.054, .849] | **[.224, .911]** |

*Note*. *M* and *SD* represent mean and standard deviation. Pearson and Spearman (^ρ^) correlation coefficients were computed to examine the relationship between patients’ alliance ratings from the initial session and therapy outcome variables, namely: change in patients’ global severity index (ΔGSI) and change in depressive symptom severity (ΔBDI), and patients’ ratings of state alliance (P_State_Alliance) and trait alliance (P_Trait_Alliance). Values in square brackets indicate the 95% confidence interval for each correlation. * indicates p < .05. ** indicates p < .01. *** indicates p < .001.

**Table 17** Correlation matrix: therapists’ alliance ratings and therapy outcome

| Variables | *M* | | *SD* | 1 | 2 | 3^ρ^ |
| --- | --- | --- | --- | --- | --- | --- |
| 1. ΔGSI | -0.150 | | 0.260 |  |  |  |
| 2. ΔBDI | -1.417 | | 5.054 | 0.482 |  |  |
|  |  | |  | [-.127, .827] |  |  |
| 3. T_State_Alliance^ρ^ | 2.000 | | 1.128 | **0.609*** | **0.626*** |  |
|  |  | |  | **[.055, .877]** | **[.082, .883]** |  |
| 4. T_Trait_Alliance | 3.750 | | 1.215 | -0.102 | 0.115 | 0.188 |
|  | |  |  | [-.639, .501] | [-.492, .646] | [-.432, .688] |

*Note*. *M* and *SD* represent mean and standard deviation. Pearson and Spearman (^ρ^) correlation coefficients were computed to examine the relationship between therapists’ alliance ratings from the initial session and therapy outcome variables, namely: therapists’ ratings of state alliance (T_State_Alliance), trait alliance (T_Trait_Alliance), and change in patients’ global severity index (ΔGSI) and depressive symptom severity (ΔBDI). Values in square brackets indicate the 95% confidence interval for each correlation. * indicates p < .05. ** indicates p < .01. *** indicates p < .001.

**Table 18** Correlation matrix: interpersonal synchrony scores and therapy outcome

| Variables | *M* | *SD* | 1 | 2 | 3 | 4^ρ^ | 5 | 6 | 7 |
| --- | --- | --- | --- | --- | --- | --- | --- | --- | --- |
| 1. ΔGSI | -0.150 | 0.260 |  |  |  |  |  |  |  |
| 2. ΔBDI | -1.417 | 5.054 | 0.482 |  |  |  |  |  |  |
|  |  |  | [-.127, .827] |  |  |  |  |  |  |
| 3. Body_sync_in | 0.172 | 0.046 | **0.606*** | -0.199 |  |  |  |  |  |
|  |  |  | **[.049, .875]** | [-.694, .423] |  |  |  |  |  |
| 4. Body_sync_anti^ρ^ | -0.107 | 0.037 | -0.028 | -0.381 | 0.462 |  |  |  |  |
|  |  |  | [-.592, .555] | [-.784, .247] | [-.153, .819] |  |  |  |  |
| 5. Head_sync_in | 0.187 | 0.035 | 0.285 | -0.373 | **0.811**** | 0.336 |  |  |  |
|  |  |  | [-.345, .738] | [-.780, .256] | **[.443, .945]** | [-.295, .763] |  |  |  |
| 6. Head_sync_anti | -0.108 | 0.016 | 0.459 | 0.119 | 0.549 | -0.007 | 0.397 |  |  |
|  |  |  | [-.156, .818] | [-.488, .649] | [-.037, .854] | [-.579, .569] | [-.229, .791] |  |  |
| 7. HR_sync_in | 0.384 | 0.133 | **0.695*** | 0.568 | 0.372 | -0.203 | 0.054 | **0.693*** |  |
|  |  |  | **[.202, .907]** | [-.009, .861] | [-.257, .779] | [-.696, .420] | [-.536, .609] | **[.198, .906]** |  |
| 8. HR_sync_anti | -0.383 | 0.135 | **0.724**** | **0.595*** | 0.096 | -0.315 | -0.120 | 0.424 | **0.764**** |
|  |  |  | **[.256, .917]** | **[.033, .872]** | [-.505, .635] | [-.753, .316] | [-.649, .488] | [-.198, .803] | **[.338, .930]** |

*Note*. *M* and *SD* represent mean and standard deviation. Pearson and Spearman (^ρ^) correlation coefficients were computed to examine the relationship between interpersonal synchrony indices with the follow-up variables of change in patients’ symptomatology over time, namely: in-phase and anti-phase body movement synchrony (Body_sync_in, Body_sync_anti), in-phase and anti-phase head movement synchrony (Head_sync_in, Head_sync_anti), in-phase and anti-phase heart rate synchrony (HR_sync_in, HR_sync_anti), change in patients’ global severity index (ΔGSI) and change in depressive symptom severity (ΔBDI). Values in square brackets indicate the 95% confidence interval for each correlation.

* indicates p < .05. ** indicates p < .01.
